# The Whale Pump: Marine Mammals Enhance Primary Productivity in a Coastal Basin

**DOI:** 10.1371/journal.pone.0013255

**Published:** 2010-10-11

**Authors:** Joe Roman, James J. McCarthy

**Affiliations:** 1 Gund Institute for Ecological Economics, University of Vermont, Burlington, Vermont, United States of America; 2 Museum of Comparative Zoology, Harvard University, Cambridge, Massachusetts, United States of America; California Academy of Sciences, United States of America

## Abstract

It is well known that microbes, zooplankton, and fish are important sources of recycled nitrogen in coastal waters, yet marine mammals have largely been ignored or dismissed in this cycle. Using field measurements and population data, we find that marine mammals can enhance primary productivity in their feeding areas by concentrating nitrogen near the surface through the release of flocculent fecal plumes. Whales and seals may be responsible for replenishing 2.3×10^4^ metric tons of N per year in the Gulf of Maine's euphotic zone, more than the input of all rivers combined. This upward “whale pump” played a much larger role before commercial harvest, when marine mammal recycling of nitrogen was likely more than three times atmospheric N input. Even with reduced populations, marine mammals provide an important ecosystem service by sustaining productivity in regions where they occur in high densities.

## Introduction

The biological pump mediates the removal of carbon and nitrogen from the euphotic zone through the downward flux of aggregates, feces, and vertical migration of invertebrates and fish [1]. Copepods and other zooplankton produce sinking fecal pellets and contribute to downward transport of dissolved and particulate organic matter by respiring and excreting at depth during migration cycles, thus playing an important role in the export of nutrients (N, P, and Fe) from surface waters [2], [3]. Perhaps because of the prevalence of this flux of zooplankton biomass and detritus, it has often been presumed that the fecal matter of top predators such as marine mammals is also lost rapidly to deep waters and the benthos [4]. Yet predators such as whales, pinnipeds, and seabirds must rise to the surface to breathe, and so may play a different role in nutrient cycling.

There is a growing body of evidence supporting the important role of large vertebrates in many ecosystem processes. Grazing animals in the Serengeti, for example, stimulate net primary productivity and carbon sequestration [5], [6]. Changes in vertebrate density and composition can have local and even global impacts: the decline of Pleistocene megafauna may have impacted methane production and thus atmospheric temperature [7]. Similarly, the removal of sperm whales from the Southern Ocean may have diminished this region's role as a reservoir for carbon [8].

Several lines of evidence indicate that most of the nitrogen released by marine mammals is expected to be in the shallower portion of their depth range: attachment to the surface for respiration, reduced metabolism at depth, physiological response to hydrostatic pressure, a decrease in glomular filtration rate and urine flow during forced diving studies, and observations of buoyant fecal plumes at the surface [9], [10], [11]. As early as 1983, Kanwisher and Ridgway noted that cetaceans could play an analogous role to upwelling, “lifting nutrients from deep waters” and releasing fecal material “that tends to disperse rather than sink when it is released.” [12] Whale foraging dives are characterized by rapid descents and ascents to reduce transit time to prey aggregations [13], [14], and high metabolic rates in gray seals while motionless at the surface support the idea that marine mammals process food during extended surface intervals following deep-water foraging [15]. Even if defecation occurred randomly, it would on average occur higher in the water column than where these animals feed, since they are unlikely to dive deeper than foraging efforts require.

**Figure 1:**
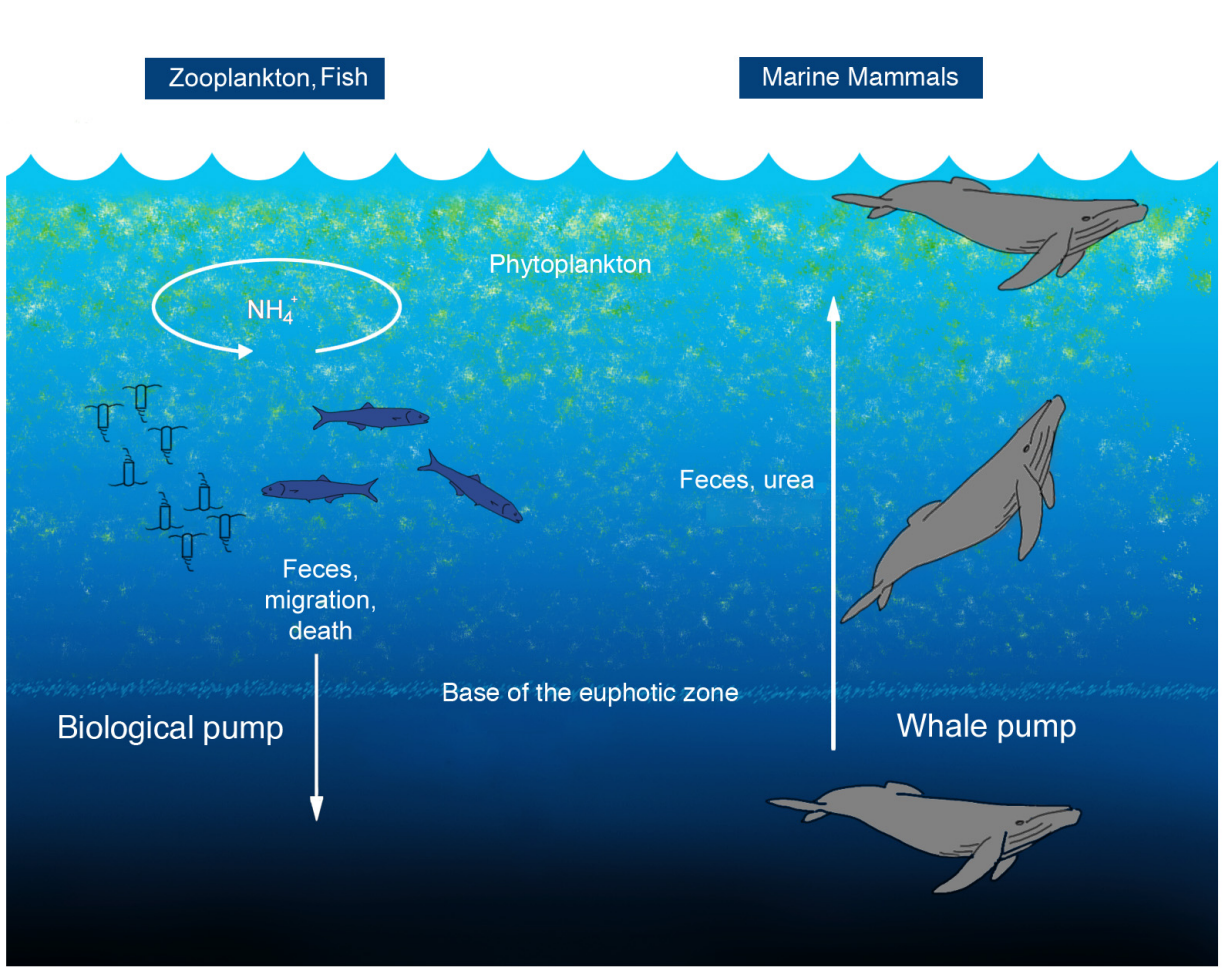
A conceptual model of the whale pump. In the common concept of the biological pump, zooplankton feed in the euphotic zone and export nutrients via sinking fecal pellets, and vertical migration. Fish typically release nutrients at the same depth at which they feed. Excretion for marine mammals, tethered to the surface for respiration, is expected to be shallower in the water column than where they feed.

Thus opposing the contribution of zooplankton, such as copepods, to the downward biological pump, cetaceans feeding deep in the water column effectively create an upward pump, enhancing nutrient availability for primary production in locations where whales gather to feed (Figure 1). Released nitrogenous compounds that can be used by primary producers are likely to remain in the euphotic zone, either as urea (the primary mammalian N-excretory product in urine), or as amino-N and NH_4_^+^ as the fecal plume material is consumed and metabolized. Pinnipeds that breed on shore and seaside ledges are also a source of nitrogenous nutrients in coastal waters [16].

We examined the relative importance of the whale pump in the Gulf of Maine, a partially isolated, highly productive basin in the western North Atlantic Ocean where nitrogen is generally considered to be the limiting nutrient for phytoplankton growth [17]. Townsend observed that the advective flux of nitrogen from deep and adjacent waters could not sustain primary production in this basin, noting that the “construction of carbon and nitrogen budgets that consider only fluxes into and out of the Gulf, and not internal recycling, will be in error” [18].

## Results and Discussion

### Field Measurements

**Figure 2:**
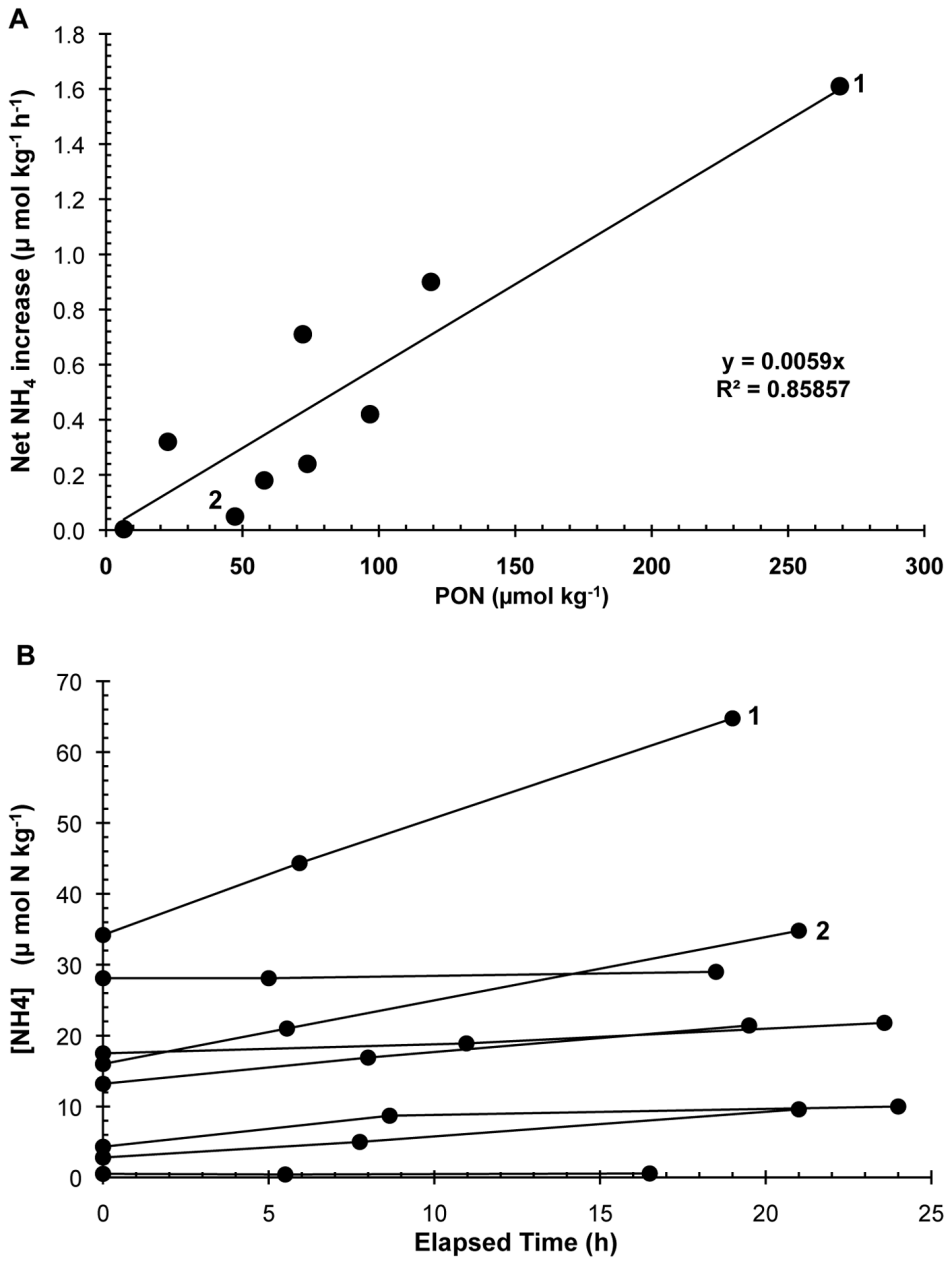
Shipboard incubation time-course experiments on Humpback whale samples collected on Stellwagen Bank, Gulf of Maine. (**a**) Net NH_4_^+^ production vs. fecal PON concentration in time course incubations of material collected in whale fecal plumes. Samples 1 and 2 had the highest initial NH_4_^+^ concentrations, yet their rates of NH_4_^+^ production ranged from the second lowest to the highest in the entire data set. (**b**) NH_4_^+^ concentration vs. incubation time.

We collected and analyzed 16 fecal plume samples during two whale-tagging cruises on Stellwagen Bank. PON concentrations of the humpback fecal plume samples were elevated by as much as two orders of magnitude above typical mixed-layer concentrations for summer in this area [19]. Concentrations of NH_4_^+^ in fecal plumes ranged from 0.4 to 55.5 µmol kg^−1^. All reference samples collected away from visible fecal plumes had concentrations <0.1 µmol kg^−1^ (the nominal limit of detection), which is typical for summer surface waters [19]. Hence, nearly all of the samples taken near whale fecal plumes had dramatically elevated NH_4_^+^. The results of shipboard incubation time-course experiments are plotted in Figures 2a and 2b. These fecal plume samples contain phytoplankton and microbes capable of utilizing NH_4_^+^. Thus any change over time would be the net difference between what was produced by microbial activity associated with the feces (presumably gut flora) and the constituent microbial plankton minus the consumption of NH_4_^+^ by plankton and microbes. No samples showed a net loss of NH_4_^+^ during these experiments.

The measured NH_4_^+^ production rates in incubated samples were strongly correlated with sample PON concentration (Figure 2a), which implicates fecal particulate material as the source of this nitrogen. The highest observed production rate was equivalent to about 50 times a typical plankton assimilation rate during summer in Massachusetts Bay [19]. Rates of increase in NH_4_^+^ show no relationship to initial NH_4_^+^ concentrations (Figure 2b), suggesting that the source is the fecal particulate material rather than another dissolved compound (amino-N or urea) that was co-released with NH_4_^+^.

### Ecosystem Effects

**Figure 3:**
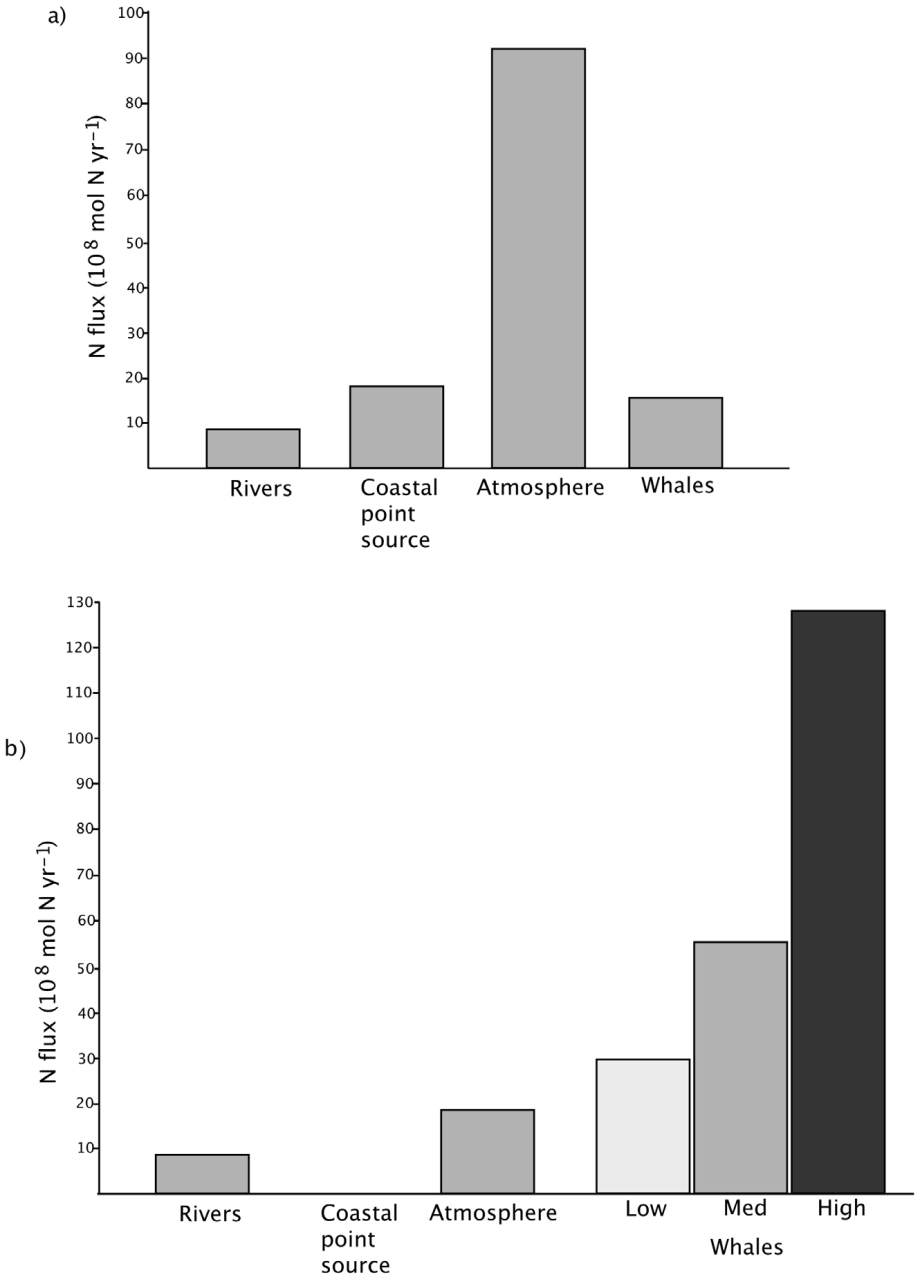
The flux of nitrogen in the Gulf of Maine (a) at present and (b) before commercial hunting. Point-source pollution, industrial emissions of nitrogen, and allochthonous sources from Townsend [18]. The range of historical estimates are adapted from Lotze [66]. Sources that are not expected to be influenced by anthropogenic change, such as offshore transport from Scotian Shelf water, are not included in this graph.

We propose that marine mammals play an important role in the delivery of recycled nitrogen to surface waters (Table 1). Over the course of a year, marine mammals release approximately 2.3×10^4^ metric tons (1.7×10^9^ mol N) per year to the surface of the Gulf of Maine, more than all rivers combined and approximately the same as current coastal point sources (Figure 3a, Table 2, [20]). Although atmospheric deposition delivers more nitrogen to the Gulf than rivers or marine mammals, it is important to note that the atmospheric source is currently much higher than the estimated preindustrial levels (Figure 3b) [21].

**Table 1:** Effect of common and historically important marine mammals on the nitrogen cycle in the Gulf of Maine ecosystem.

Species	N excreted (kg day^−1^)	Population (*N*)	N flux (10^8^ mol N yr^−1^)
Cetaceans			
Baleen			
Right whale	15.9	345	1.2
Humpback whale	9.42	902	1.8
Fin whale	15.0	2,065	6.7
Sei whale	8.32	91	0.16
Minke whale	2.94	3,497	2.3
Toothed			
Pilot whale	0.63	219	0.036
White-sided dolphin	0.15	20,400	0.78
Common dolphin	0.09	139	0.0034
Harbor Porpoise	0.05	89,700	1.2
Pinnipeds			
Harbor seal	0.09	99,340	2.4
Gray seal	0.22	1,731	0.10
Total			16.7

Total annual nitrogen released is 365 x N excreted day^−1^ for resident toothed whales and pinnipeds; for baleen whales, which migrate seasonally out of the study area, the total nitrogen released is expected to be 83% of annual excretion [48].

**Table 2:** Contemporary nitrogen flux in the Gulf of Maine.

Source	N flux per year (10^8^ mol N)
**Biological**	
Cetaceans	14
Pinnipeds	2.5
Seabirds	1.2–2.3
**Influx**	
Offshore	1,479
Rivers	8
Coastal point sources	18
Atmosphere	93
**Loss**	
Denitrification	331
Burial	44

Influx and loss from Townsend [18].

Coastal point sources from Sowles [20].

**Figure 4:**
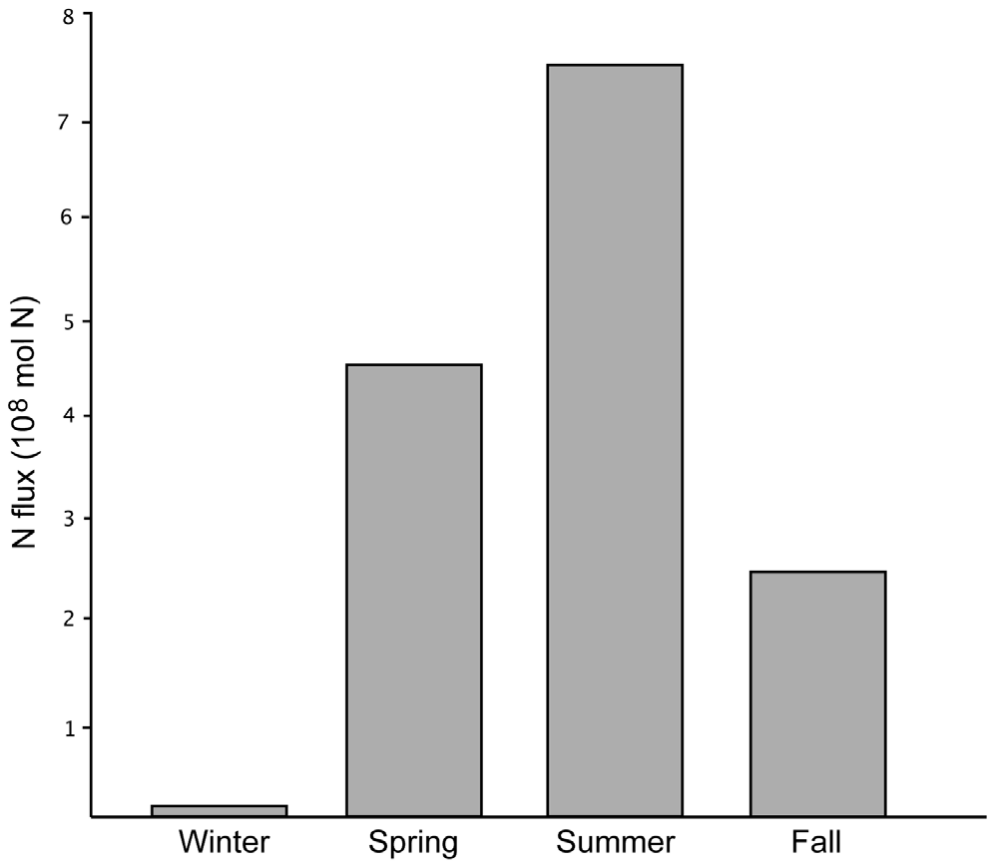
The role of cetaceans in the nitrogen cycle by season. Seasonal estimates based on the percentage of total consumption in the Gulf of Maine [24].

The release of nutrients at the ocean surface is a pattern common to many air-breathing vertebrates, however, in the Gulf of Maine, and presumably in many other systems, it is dominated by whales, especially baleen whales. Currently cetaceans deliver approximately 77% of the nutrients released to the gulf by mammals and birds (Table 2); their biomass in the North Pacific and Southern Oceans indicate that they also play a dominant role in these systems [22], [23]. For some marine ecosystems it may be appropriate to expand this term beyond one that emphasizes whales to acknowledge greater importance of pinnipeds or seabirds. In the gulf, the whale pump will be most active in spring and summer, when feeding whales are present and when nitrate levels are low (Figure 4). Concentrations are ∼8 µmol kg^−1^ in winter but approach undetectable levels in summer [18]. Kenney et al. have estimated that 30% of the annual prey consumed by cetaceans in the Gulf of Maine occurs in spring and 48% in summer [24]. Surface excretion may extend seasonal plankton productivity during these seasons, after a thermocline has formed. The effects of the pump are also expected to be much greater in highly productive areas such as Stellwagen and Georges Banks and the Bay of Fundy, where diving and surfacing transcends warm-season stratification and can markedly increase surface nitrogen levels.

The whale pump provides a positive plankton nutrition feedback. On Stellwagen Bank, humpback whales bottom feed on sand lance (*Ammodytes* spp), especially at night when these forage fish burrow into the sandy substrate [25]. In the Grand Manan Basin, right whales feed beneath the thermocline, on concentrated bands of diapausing copepods, in direct proportion to the abundance and quality of food available [14], [26]. The density of copepods in this layer is orders of magnitude greater than average estimates of water-column prey density [27]. The average dive depth (113–130 m) for right whales is strongly correlated with peak prey abundance (fifth copepodites of *Calanus finmarchicus*) and the thermocline [14]. Fin whale foraging dives often exceed 100 m to locate dense concentrations of euphausiids [13].

Not all feeding occurs along or below the pycnocline. Right whales surface feed on copepods in Cape Cod Bay and the Great South Channel in the spring [28]. On Stellwagen, humpbacks tend to surface feed during daylight hours, when their prey is most abundant in the upper portion of the water column [25]. Several species have diel patterns in foraging behavior: sei whales feed on aggregations of *C. finmarchicus* when they migrate to the surface at night, reducing transit time for the whales and maximizing foraging efficiency [29]. Although the upward movement of nutrients is essential to our conception of the whale pump, the feeding of marine mammals at the surface, especially on prey that migrate across the pycnocline themselves, and the subsequent excretion of nutrients at the surface are important parts of the overall pattern of the pump.

Because of their large size and the high energetic cost of foraging, baleen whales require dense patches of food [13]. Production of phytoplankton stocks that support copepods, euphasiids, and fish consumed by whales will benefit most immediately from the release of nitrogenous excreta in nutrient-limited waters during stratified summer conditions. The whale pump could also reinforce the aggregative behavior and cooperative foraging of some cetaceans. The predictability of finding food in regions of high productivity is critical to individual survival and reproductive success: many species return to the same locations year after year, using the same feeding grounds across generations [30], [31]. Another possible concentration-enhancing mechanism of the whale pump is the attraction of zooplankton to fecal material. The initial observation that led Hamner and Hamner to study the use of scent trails by zooplankton was an aggregation of copepods on the regurgitated meal of a seasick dive-boat tender [32]. At least one of the fecal plumes we collected—suspended just below the surface, about the size of our inflatable sampling boat, and the color of oversteeped green tea—had high numbers of copepods. Consumption of the fine particulate fraction in the fecal plume by zooplankton would provide further nutrition for the lower trophic levels that nourish these mammals.

Any attempt to study the role of marine mammals in coastal ecosystems must consider that many species now occur only in remnant populations, drastically reduced by commercial exploitation, incidental mortality, and habitat destruction (Figure 3b). Three species of mammals (sea mink, Atlantic walrus, and possibly Atlantic gray whale) are now extinct or absent in the Gulf of Maine, along with several marine birds, including the great auk. In the Bay of Fundy, humans have reduced the biomass of the upper trophic level of vertebrates by at least an order of magnitude [33]. One unanticipated consequence of this depletion of deep-diving mammals is a likely decline in the carrying capacity for higher trophic levels in coastal ecosystems.

Looking beyond the Gulf of Maine, it is important to consider the roles of present and past stocks of large air-breathing predators in the nutrient cycle of marine ecosystems. In the North Pacific, whale populations consume approximately 26% of the average daily net primary productivity; pre-exploitation populations may have required more than twice this sum [34]. Might primary productivity have been higher in the past as a result of a stronger whale pump? One recent study provides evidence that phytoplankton abundance has declined in 8 of 10 oceanic regions over the past century, and the authors suggest that this can be explained by ocean warming over this period [35]. Yet declines in both the Arctic and Southern Ocean regions, areas with especially high harvests of whale and seal populations over the past century, are in excess of the mean global rate. Full recovery from one serious anthropogenic impact on marine ecosystems, namely the dramatic depletion of whale populations, can help to counter the impacts of another now underway—the decline in nutrients for phytoplankton growth caused by ocean warming. The whale pump may have even played a role in helping to support a greater number of apex consumers. In the Southern Hemisphere, Willis has noted that a decrease in krill abundance followed the near elimination of large whales [36]. He hypothesized that one factor in this counterintuitive decline is a shift in krill behavior. Another factor could be the diminished whale pump, which would have affected productivity by reducing the recycling of nutrients to near-surface waters: Smetacek and Nicol et al. have shown that whales recycle iron in surface waters of the Southern Ocean [23], [37]. The fertilization events of the whale pump can apply to nitrogen, iron, or other limiting nutrients.

These findings have important implications for the management of ocean resources. As marine mammal populations recover, it has been suggested that whales and other predators should be culled to limit competition with human fishing efforts, an idea that has been championed to challenge international restrictions on whaling [38]. Yet no data have been forthcoming to support the logic of this assertion. Furthermore, recent studies suggest that marine mammals have a negligible effect on fisheries in the North Atlantic [39], [40]; simulated reductions in large whale abundance in the Caribbean did not produce any appreciable increase in biomass of commercially important fish species [41]. On the contrary, marine mammals provide important ecosystem services. On a global scale, they can influence climate, through fertilization events and the export of carbon from surface waters to the deep sea through sinking whale carcasses [42]. In coastal areas, whales retain nutrients locally, increasing ecosystem productivity and perhaps raising the carrying capacity for other marine consumers, including commercial fish species. An unintended effect of bounty programs and culls could be reduced availability of nitrogen in the euphotic zone and decreased overall productivity.

## Methods

### Ammonium analysis

An important question in this research was whether elevated NH_4_ could be detected in whale fecal plumes, and whether rates of NH_4_^+^ production could be measured when freshly sampled feces are held in experimental chambers in the shipboard laboratory. Humpback whale fecal plumes were sampled with a 30-cm diameter, 150-µm mesh plankton net from small boats engaged in whale-tagging operations on Stellwagen Bank during July 2008 and 2009. The large greenish plumes, typically suspended just below the surface and at times as big as the collecting boat, were visibly heterogeneous and did not allow for quantitative sampling. Surface-water controls away from visible fecal plumes were collected both in close proximity (∼20 m) to groups of surfacing whales and distant (>1 km) from any visible activity.

One-liter samples were placed in a cooler and returned to the support ship (NOAA Ship *Nancy Foster*) within 1–6 hours of collection, at which time a 200-ml aliquot of the fecal suspension was filtered (combusted Whatman GF/F). The filtrate was analyzed for initial NH_4_^+^-N concentration [43]. The filter was dried at 50°C, then sealed in a glass vial and retained for later particulate organic nitrogen (PON) analysis onshore [44]. The remaining unfiltered sample was placed in a dark refrigerator (12°C) to monitor changes in NH_4_^+^ over time. (Mean surface water temperature during the study period was ∼18°C.)

At approximately 10 and 20 hours from the time the samples were onboard, subsamples were drawn from the refrigerated sample, filtered, and the filtrate was analyzed for NH_4_^+^-N concentration. In addition, single point NH_4_^+^-N and PON determinations were made on the control water samples described above, as well as samples from eight additional distinct fecal plumes sampled during this period and a similar operation in July 2008. Extremely dense aggregations of copepods were observed in a few fecal samples. We were unable to satisfactorily remove animals in these samples for analysis of fecal PON, and thus data from these samples are not reported here. We did not determine if the copepods were coprophagous.

### Marine Mammal Consumption

To calculate the effect of marine mammals on the nitrogen cycle, we used estimates of daily consumption employing standard metabolic models scaled for assimilation, activity, and migratory fasting. This consumption rate has traditionally been estimated as 2–3% of body mass for rorqual whales, representing a daily average for summer consumption in Antarctica [45]. We employed more conservative estimates, as considered by Barlow and colleagues [46], using mass (*M*) to calculate the basal metabolic rate (*BMR*), where BMR  = 293.1 *M* ^0.75^. Rather than relying on a factor of 2.5 x BMR to calculate the field metabolic rate (FMR) we used 3 x BMR, in light of recent studies by Kjeld and colleagues, who derived consumption rates of 3.5% per day for fin whales and 4.6% for sei whales—about 30% higher than previously estimated [47]. Lockyer also found higher levels of consumption, calculating that baleen whales increase consumption rates ten fold in the summer [48]. The average daily ration was calculated as FMR divided by (0.8[3900*Z* + 5450(1–*Z*)]), where *Z* is the fraction of crustaceans in the diet [46]. Values for *Z* are from the dietary composition table in Kenney et al. [24]. See Table 3 for daily consumption rates.

**Table 3:** Body mass and consumption rates for cetaceans and seals in the Gulf of Maine.

Species	Body mass (kg)	Percent of zooplankton in diet	Wet weight consumed (kg day^−1^)
Cetaceans			
Baleen			
Right whale	40,000	100	797
Humpback whale	30,408	5	471
Fin whale	55,590	10	751
Sei whale	16,811	100	416
Minke whale	6,566	5	149
Toothed			
Pilot whale	850	0	32
White-sided dolphin	120	0	7.3
Common dolphin	65	0	4.6
Harbor porpoise	31	0	2.6
Pinnipeds			
Harbor seal	67	0	4.6
Gray seal	160	0	11

We employed an average daily consumption rate of 6.9% for seals in the Gulf of Maine, based on data from gray seals collected by Sparling et al. [15]. This aligns well with data from other pinnipeds, such as sea lions, which require daily consumption of between 5% (adult males) and 13% (young females) of their body mass, with lactating females increasing their consumption by 70% [49]. Carlini et al. estimated a consumption rate of 6.8% during the post-breeding aquatic phase for southern elephant seals [50].

### Marine Mammal Nitrogen Excretion

Fish and crustaceans such as euphasiids are approximately 15% protein [45] (about 17% nitrogen by weight) or 2.5% nitrogen. Nitrogen consumption  =  feces + urine + storage. Feces and urine are egested; stored nitrogen is retained for growth, energy reserve, eggs, sperm, and embryos. We assume that approximately 80% of ingested nitrogen is metabolized and 20% is retained [51]. Although the great majority of fecal matter is expected to stay in the euphotic zone, we employed this conservative estimate to account for the fact that no quantitative analysis has been performed to account for potential sinking. Although prey consumption and body weight vary according to age and reproductive status, we employed average adult weights for all marine mammals.

Pinnipeds excrete approximately 87% of ingested nitrogen [16], [52]. We employed an estimate of 80% to account for potential exported nitrogen. We recognize that seal feces can be important to the coastal ecosystem, but assume that the amount retained by terrestrial systems would be negligible in relation to the total nitrogen flux. Even during the breeding period, pinnipeds such as sea lions spend more than 80% of their time at sea [53]. Rookeries are rarely far from the sea, and it is assumed that most nutrients are returned to the ocean during storms [16]. Approximately 3% of the excretion from pinniped colonies is expected to be volatilized as NH_3_ into the atmosphere [16], with some of this nitrogen returned to the sea via wet atmospheric deposition.

Urinary nitrogen from marine mammals would disperse diffusively and advectively, and the amount released would be difficult to sample quantitatively. Particulate and dissolved nitrogen associated with flocculent fecal plumes can, however, be sampled because the plumes are visible from ships. Microbial proteolitic and deaminating processes will liberate NH_4_^+^ from the released particulate material, and these processes may have begun in the animal's gut.

### Seabirds

Seabird estimates were unavailable for the entire Gulf of Maine. Huettmann estimated that the total marine food consumption of the 10 most common seabirds along the western Scotian Shelf was approximately 84,000 tons per year [54]. As the Scotian Shelf forms the eastern boundary of the Gulf of Maine, we used this annual consumption estimate of 0.87 tons km^−2^ yr^−1^ to determine the total effect of seabirds on the nitrogen cycle in the Gulf of Maine. Powers & Backus estimated an annual consumption rate of 1.6 tons km^−2^ yr^−1^ for the seabirds of Georges Bank [55]. We employed these two rates to estimate a reasonable range of the role that seabirds play in this basin.

For seabirds, foraging effort may be targeted at the zone below the thermocline [56], and nutrient cycling is expected to be quick. In birds, nitrogen is excreted primarily as uric acid, which is unstable in seawater, undergoing rapid conversion to urea [57]. We estimated that approximately 80% of nitrogen consumed was excreted at the surface, with 20% stored for fat and reproduction or exported to terrestrial systems and the seafloor. The entire area of the Gulf of Maine is 1.03×10^5^ km^2^ [18], yielding a total nitrogen flux of 1.2–2.3×10^8^ mol N yr^−1^, or about 10% of the current nutrient contribution from marine mammals.

### Body Mass, Residence Time, and Population Size for Marine Mammals

Body mass is from Trites and Pauly [58], using mean mass of males and females assuming a 1∶1 sex ratio. Right whale body mass is from Kenney et al [24]. Population size for cetaceans is also from Kenney et al., employing an average of the summer and spring estimates of abundance, except for humpback whales [59], harbor porpoises [60], white-sided dolphins [61], and gray and harbor seals [61], [62]. Right and fin whale populations are from NOAA stock assessments [61] Estimates for fin whales come from a survey conducted in 2006 from the southern Gulf of Maine to the Gulf of Saint Lawrence. Although part of this survey took place outside of our study area, the numbers are lower than previous studies for just the Gulf of Maine. We applied this abundance estimate as a reasonable, and conservative, estimate. Seal estimates are also probably conservative: many harbor seals are year-round residents, and we only account for the spring and summer seasons when they are pupping along the Maine coast (assuming that 50% of their yearly ration comes from the gulf). Both harbor and gray seal populations have likely grown since the last estimates were made (harbor seals in 2001, gray seals in 1999).

Total annual nitrogen flux was estimated as the product of the mean annual flux (365 x N excreted day^−1^) and the estimated abundance of each species. For baleen whales, which migrate outside of the study area, we used Lockyer's estimate that 83% of the annual intake occurs in summer feeding areas [46], [48].

### Seasonal variation

We expect seasonal variation in feeding, as has been observed in captive adult gray seals [63] and many other marine mammals [64]. Periods of fasting in pinnipeds, for example, are assumed to be balanced by periods of more intensive feeding over the course of the year [65]. Because feeding is likely to decline in the winter, we suspect that our estimates are conservative for the many of the organisms included in this study.

### Historic Estimates

We used data from Lotze et al. [66] to estimate historical numbers of cetaceans in the Gulf of Maine. Large whales in Massachusetts Bay are 10% of their historical numbers and small cetaceans 50%. In the Bay of Fundy, large whales were estimated to have a relative abundance of 45% compared to pre-exploitation numbers and small cetaceans 50%. We took estimates for Massachusetts Bay as the upper end for past population sizes and estimates from the Bay of Fundy in the lower end. It is worth noting that several ocean-wide studies support the higher end of this range [67], [68]. As a medium estimate, we took an approximate average of these percentages, assuming that large whales constitute 25% of historical numbers and small cetaceans 50%.
